# Development of a survey and worry score to evaluate physician burnout and wellness interventions during COVID-19 in the Rio Grande Valley: A pilot study

**DOI:** 10.1371/journal.pone.0342993

**Published:** 2026-03-20

**Authors:** Victoria Jacobsen, Ericka A. Vazquez, Lauren Herrera, Ruth Escalera, Daniel Salinas, Bharathi S. Gadad, Juan Carlos Lopez-Alvarenga, Kelsey Potter-Baker

**Affiliations:** 1 University of Texas Rio Grande Valley, School of Medicine, Edinburg, Texas, United States of America; 2 Division of Population Health & Biostatistics, School of Medicine, University of Texas Rio Grande Valley, Edinburg, Texas, United States of America; 3 University of Texas Rio Grande Valley, School of Medicine, Neurology and Behavioral Health Integrated Service Unit, Edinburg, Texas, United States of America; College of Nursing, Qassim University, SAUDI ARABIA

## Abstract

**Background:**

Current research on physician burnout is limited in rural communities and physicians caring for the medically underserved. This study aimed to create an instrument to quantify the rate of physician burnout during the COVID-19 pandemic and identify effective coping interventions in an underserved mixed rural-urban region along the Texas–Mexico border.

**Methods:**

We conducted a regional study in a sample of US physicians using a structured questionnaire developed by a multidisciplinary group. Thirty-one participants were contacted over a five-week period between December 2022 to January 2023. Participants self-selected based on their experiences of burnout during various phases of the COVID-19 pandemic (e.g., Initial Surge, Lock Down, Adjustment Period, Vaccine Distribution, Delta Variant, Booster Available). A 5-point Likert scale was used to assess and rank the perceived effectiveness of services employed to cope with burnout and its negative implications.

**Results:**

We found that 43% of participants experienced burnout during the COVID-19 pandemic, with flexible schedules and personal wellness being the top alleviators of burnout. The survey showed good internal consistency and reliability (Cronbach’s alpha coefficient = 0.7680), McDonald’s omega was 0.80, and identified three “worry types” through latent factor analysis. Worry types included: (1) physicians with a desire to self-preserve but showed negative coping, (2) physicians with a desire for occupational harmony but with concerns about patient safety, and (3) physicians with concerns about personal safety and their mortality potential. Additionally, latent factor analysis showed that 9 questions from the survey could be used to create a Worry Score to classify burnout and coping strategies related to the COVID-19 pandemic.

**Conclusion:**

These findings suggest that healthcare administrators and policymakers should prioritize implementing and promoting flexible work schedules and opportunities for personal time as part of comprehensive strategies to improve physician wellness and mitigate burnout. Additional research is needed to determine the effectiveness of the interventions in addressing physician burnout.

## Introduction

The Coronavirus Disease 2019 (COVID-19) pandemic highlighted several critical concerns within the American healthcare system, including resource allocation, healthcare worker safety, and systemic inefficiencies [1–5]. Physician burnout, or “long-term stress reaction marked by emotional exhaustion, depersonalization, and a lack of sense of personal accomplishment”, emerged as a significant concern during the pandemic, primarily due to increased workloads, emotional strain, and the rapid adaptation to new clinical protocols [6–11]. Studies have suggested that physician burnout can result in early career departure, increased medical errors, poorer continuity of care, and ultimately threaten patient safety [12–23]. For example, Shanafelt et al. (2010) reported a 45% higher incidence of medical errors among burned-out physicians [22]. In addition, physician burnout has been associated with impaired critical thinking function [14,24]. According to a 2022 Medscape report, 58% of physicians report experiencing burnout, with 68% saying it negatively affects their relationships and 54% noting a severe impact on their personal and professional life. Similar trends have also been noted after the COVID-19 pandemic [25–28].

Studies have found that workplace activities significantly impact burnout levels [29–34]. For example, the introduction of computerized order entry has been associated with a 29% increase in burnout. Additionally, the shift in workplace activities, with physicians now spending two hours on electronic health records (EHR) and desk work for every hour of direct patient interaction, exacerbates the issue of burnout [35–38]. Additionally, other studies have found organizational issues such as long work hours, overnight calls, and lack of leadership support have been identified as significant risk factors for physician burnout [19,39,40]. Effective solutions to burnout often combine personal wellness activities, such as stress-management techniques and exercise with organizational changes, including the reduction of work hours and optimization of EHR systems [41–43]. Further, recognizing the critical impact of physician burnout, the World Health Organization recently defined it as an organizational issue requiring an organizational solution, underscoring the importance of systemic interventions over individual ones [44–46].

To institute organizational solutions to burnout, it is critical to understand the localized triggers of burnout. Unfortunately, readily available burnout surveys have very limited and reported use in rural populations [13,47–49]. Further, while aspects of physician burnout have been well described from the COVID-19 pandemic, physicians practicing in rural and medically underserved areas may have different causes of burnout and require different systemic interventions [18,24,41,50–53]. In particular, border regions and urban-rural communities often face unique challenges, such as limited healthcare resources, high patient loads, and socioeconomic disparities, necessitating tailored strategies to address physician burnout [51,54]. For example, the Rio Grande Valley (RGV) is a mixed urban-rural community located on the Texas-Mexico border and is home to many unique conditions that increase the risk of physician burnout. Specifically, in 2013 it was estimated that over half of the RGV population is uninsured, not including a large and ever-evolving population of undocumented immigrants [55]. Further, primary and specialty physician shortages have been reported throughout the state of Texas, with the RGV region showing extreme shortages [56,57]. For example, in 2021, Texas county health records indicated that there were only 62 primary care physicians per 100,000 residents in the RGV, compared to the national average of 91 per 100,000. Such complex work environment for RGV physicians may contribute to different degrees of physician burnout, with the COVID-19 pandemic exaggerating it due to increased patient loads, heightened emotional stress, and the rapid implementation of new clinical protocols and telehealth services.

Given the unique burden and factors influencing physician burnout in rural areas and limited assessment tools, this study primarily sought to build a new survey instrument to measure burnout experienced by physicians working in rural and medically underserved populations before and during the COVID-19 pandemic. Other burnout inventory tools, such as the Copenhagen burnout inventory or Maslach burnout inventory have inherent limitations for use in our settings. The Maslach burnout inventory measures three different constructs of burnout that limit interpretability, due to possible bias [58,59]. In addition, the Copenhagen burnout inventory has been suggested to be subject to cultural bias, an influence that is exaggerated in a rural and culturally diverse area [60–65]. Further, each of these surveys would have increased the burden of the participant when evaluating burnout in a cross-sectional study design at multiple phases of the COVID-19 pandemic, given the length of the inventory instrument.

We desired to identify if specific worry factors (worry score) contributing to burn out could be identified in our population, which would allow for development of organizational solutions. Specifically, we sought to evaluate the frequency and trends of burnout, identify common wellness practices that served to prevent or mitigate burnout and evaluate how wellness interventions may be tailored for our underserved region. Our study also aimed to evaluate the development of burnout and worry factors during different periods of the COVID-19 pandemic (i.e., initial surge, lockdown phase, vaccine distribution period, etc.), to provide more specific associations with experienced burnout. Our main objectives included proof of survey internal consistency, dimensionality, and characterization of personal perception regarding burnout associated with specific stressors. We hypothesized that we would be able to identify unique coping mechanisms affecting burnout in the RGV and suggest potential systemic changes to address the problem in rural areas.

## Materials and methods

### Study design and setting

A structured, web-based questionnaire was designed to assess perceived burnout status as defined by the Agency for Healthcare Research and Quality (2022) in a participant population of Doctor of Medicine (M.D.) physicians across the Rio Grande Valley region (Appendix A). We used nine questions (described in Tables 3 and 4, below) on a 1–5 Likert scale; higher scores indicate greater endorsement of the item. The Worry score was computed with the sum of the 9 items, with a maximum of 45 points. Q65 [How often did you find yourself relying on positive coping mechanisms during the selected time course period?] was conceptually positive coping and opposite direction loading, so we implemented reverse coding to maintain higher scores to reflect greater worry. Study data were collected and managed using REDCap electronic data capture tools hosted at University of Texas Rio Grande Valley (UTRGV) [66,67]. REDCap (Research Electronic Data Capture) is a secure, web-based software platform designed to support data capture for research studies, providing 1) an intuitive interface for validated data capture; 2) audit trails for tracking data manipulation and export procedures; 3) automated export procedures for seamless data downloads to common statistical packages; and 4) procedures for data integration and interoperability with external sources.

To ensure content validity, the questionnaire was developed, following similar methodology of other studies [68,69], through a collaborative process involving feedback and rigorous review from five medical students, three M.D. physicians, two Doctor of Philosophy (Ph.D.) researchers, and two other Ph.Ds, selected for their expertise in clinical practice, research methodology, and statistical analysis. Their input helped contribute to the questionnaire’s validity, reliability, and comprehensive coverage of burnout indicators. The questionnaire was distributed online using REDCap to avoid in-person interactions and make it easier to tailor specific needs to our study, as paper surveys are utilized less and result in reduced response rates. The following demographic data were collected as part of the questionnaire: ethnicity, biological sex, marital status, medical specialty, workplace setting, and whether medical training was completed in the United States (US MD, or US Medical Degree) or internationally (IMG or International Medical Degree). These variables were selected to explore potential demographic influences on burnout rates among physicians. Within our questionnaire, we included identifying the presence or absence of burnout during a specific time-period in the pandemic, defined by six Time Course Period variables (Table 1) that captured variations in pandemic intensity and associated stressors. The online questionnaire categorized coping strategies as either “negative” or “positive” based on established psychological frameworks [70–72]. Negative coping strategies refer to behaviors such as avoidance or substance use, while positive coping strategies include problem-solving and seeking social support. Our goal was to identify potential modifications in burnout during COVID-19 development, response, and medical discovery. This would help identify specific periods where interventions might be most needed. Survey questions also included language referencing standardized burnout surveys to increase internal consistency [13,38,73–76]. The questionnaire and study design were reviewed and approved by the University of Texas Rio Grande Valley Institutional Review Board (IRB) (IRB-22–0116). Ethical considerations, including informed consent and confidentiality of participant data, were strictly adhered to throughout the study.

**Table 1:** Defined Time Course Period variable options for use within the web-based questionnaire regarding the COVID-19 pandemic.

Defined Time Course Period	Time Period
Initial Surge	February 2020 – March 2020
Lockdown	March 2020 – May 2020
Adjustment Period	June 2020 – November 2020
Vaccine Distribution	December 2020 – June 2021
Delta Variant	June 2021 – September 2021
Booster Available	October 2021 – December 2021

### Participants

Participants who worked within the University of Texas Health Rio Grande Valley medical system in either the inpatient, outpatient, or both settings were invited to take the structured 5-point Likert scale questionnaire (answered only once) over a five-week period from 12/2022–01/2023. For recruitment, physicians were contacted directly three times via listed work emails and indirectly through weekly invites included in the Employee E-bulletin. Prior to taking the survey, all physicians provided electronic consent to participate in the study. Eligibility criteria for participation included 1) an adult over 18 years old, 2) a physician holding an M.D. degree, 3) actively practicing within any medical specialty in the two years between 01/2020 and 12/2021, and 4), informed consent to participate in the questionnaire.

### Statistical methods

Descriptive statistics provided a summary of participant characteristics with calculated frequencies (percentages) for categorical variables and mean (standard deviation) for continuous variables. The missing values by item were common because respondents were not required to answer all questions. The analyses used available data; a description of each item was summarized in Supplementary Table 1. The Worry Score was calculated as the sum of the nine items using available responses. No imputation was performed, given the pilot sample size. The prevalence of self-reported career burnout (Q13. Have you ever experienced burnout?), and COVID-19 pandemic burnout (Q14. Did you experience burnout during COVID-19?) was determined by calculating the proportion of participants reporting burnout at any point specifically during the defined COVID-19 periods. We measured the perceived burnout status and used it for known-group validation. The internal consistency across questionnaire items was assessed with Cronbach’s alpha coefficient where values indicated internal reliability, and higher values suggested agreement between standardized items (e.g., mean of 0, and standard deviation of 1) [77]. The factor analysis (see below) revealed item redundancy and unequal loadings; McDonald’s omega was calculated under a single-factor model using maximum likelihood. Additionally, the average inter-item correlation and 95% confidence interval (95% CI) were calculated through bootstrapping with 100 repetitions to provide a more robust estimate of the correlation and its uncertainty, particularly in the context of this study’s small sample size and non-normality of the data or correlation coefficient [78]. Sensitivity analyses were conducted by assessing the influence of individual items on the coefficient through item omission. To explore dimensionality, latent factor analysis was performed using Principal Component Analysis (PCA) on nine questionnaire items [79,80]. In this process, we identified the underlying factors by examining factor loadings associated with the items, providing a composite score that reflects the participant’s level of worry (i.e., a total Worry Score) based on the principal components identified. Particularly, Kaiser-Varimax rotation revealed three factors with factor loadings above 0.4, explaining 80% of the total variation [81]. The three factors identified included [1] patient safety and occupational harmony, [2] negative coping mechanisms and [3] personal safety.

Due to the robustness against non-normally distributed data, a Wilcoxon Rank-Sum test was used to contrast the three factor’s Worry Score against the presence of known-group validity burnout (yes/no), biological sex (Male/Female), and country of medical training completion (US/International Medical Graduate (IMGs)). The sensitivity of the Worry Score in detecting differences between groups was evaluated across different Time Course Periods of COVID-19 pandemic development using the Kruskal-Wallis test [82], which is designed for group comparisons of non-parametric data. Statistical analyses were performed using Stata v18 (StataCorp, College Station, TX), and McDonald’s omega with R (version 4.5.2., 2025) package **psych**.

## Results

### Participants and descriptive data

Of the 224 physicians contacted, 33 physicians participated, and 31 physicians met all eligibility criteria for inclusion within the dataset analyses, resulting in a 14.7% response rate (Table 2). The low response rate may limit the generalization of findings, but it is consistent with the typical response rates for physician surveys [34,83,84]. As seen in Table 2, demographics included the following: 61% Hispanic or Latinx, 35% female, 61% male, 4% preferred not to answer biological sex, 77% married, 84% in academia, 55% US M.D. graduates, and 45% IMGs. Ages ranged from 38–69 years old, with a mean age of 43.

**Table 2:** Demographics of Participants.

Demographic Variables	Descriptive statistic
**Age years mean (SD)**	43.38 (12.74)
**Sex n (%)**	
Male	19 (61%)
Female	11 (35%)
Prefer not to say	1 (3%)
**Job Setting n (%)**	
Academic Medicine	26 (84%)
Hospital Employed	2 (6%)
Private Practice	1 (3%)
Prefer not to say	2 (6%)
**Marital Status n (%)**	
Married	24 (77%)
Unmarried	3 (10%)
Divorced	2 (6%)
Prefer not to say	2 (6%)
**Medical School Setting n (%)**	
US MD	17 (55%)
International Medical Graduate (IMG)	14 (45%)
**Ethnicity**	
Hispanic/ Latinx	19 (61%)
Non-Hispanic White	6 (19%)
Non-Hispanic Black	1 (3%)
Other ethnicity	5 (16%)
**Specialty n (%)**	
Family Medicine	6 (19%)
Internal Medicine	6 (19%)
Pediatrics	4 (13%)
Surgery	4 (13%)
OB/GYN	3 (10%)
Other	6 (19%)
Prefer not to say	1 (3%)

### Questionnaire results and internal consistency reliability

We observed that the prevalence of burnout perception at any point during participants medical career was self-reported by 63% of participants. Specifically, 43% of participants indicated experiencing burnout during the COVID-19 pandemic from January 2020 to December 2021. Descriptive statistics for the nine Worry Score items are in Supplementary Table 1. The response rates in participants with positive perceived burnout varied across items (48–68%). The mean scores were high (3.6 to 4.5) on a 5-point scale. Participants could select “N/A” for individual items, meaning the absence of endorsed worry. Therefore, “N/A” was coded by zero, not a Likert-scale response, and included in the total Worry Score. We did not have missing values in the sample. We evaluated the reliability or internal consistency of the questionnaire using Cronbach’s alpha coefficients. Table 3 displays Cronbach’s alpha coefficients, valid observation counts, and collective influence on the questionnaire. Questions were optional for respondents; thus, the total sample size varied per question. The estimated correlation among the items within the same domain stood at 0.87 (Cronbach’s alpha = 0.76, 95% CI: 0.48, 1.0), with an average inter-item correlation of 0.26 (95% CI: 0.07, 0.44), indicating a measure of internal consistency among the questions. The estimated McDonald’s omega was 0.80, complementing the alpha values and giving conceptual appropriateness.

**Table 3:** Cronbach’s Alpha Analysis with individuals with perception of burnout.

Question Item	N	Positive or Negative Influence	Item-Test Correlation	Average Inter-Item Correlation	Cronbach α
Survey #24 (Q74) On a scale of 1–5, how often did you find yourself relying on these negative coping mechanisms during the selected Time Course Period?	17	–	0.2110	0.3150	0.7862
Survey #27 (Q82) On a scale of 1–5, how strong was your desire to leave the field of medicine during the selected Time Course Period?	13	–	0.6731	0.2413	0.7178
Survey #21 (Q65) On a scale of 1–5, how often did you find yourself relying on positive coping mechanisms during the selected Time Course Period?	21	+	0.4192	0.3022	0.7760
Survey #18 (Q57) On a scale of 1–5, how much did you feel MOST NEGATIVELY affected in your relationships, personal or work-related during the selected Time Course Period?	19	+	0.8173	0.2190	0.6917
Survey #15 (Q54) On a scale of 1–5, how much did you feel MOST NEGATIVELY affected the quality of care you provided to patients during the selected Time Course Period?	18	+	0.6520	0.2467	0.7238
Survey #12 (Q51) On a scale of 1–5, how much did you feel MOST affected in your ability to ensure patient safety during the selected Time Course Period?	18	+	0.7675	0.2150	0.6866
Survey #9 (Q43) On a scale of 1–5, how much did you feel MOST negatively affected in your typical work life during the selected Time Course Period?	21	+	0.7033	0.2328	0.7082
Survey #3 (Q25) On a scale of 1–5, how much did you feel MOST burnt out during the selected Time Course Period?	18	+	0.6565	0.2397	0.7161
Survey #6 (Q35) On a scale of 1–5, how much did you feel MOST concerned about your safety during the selected Time Course Period?	21	+	0.4446	0.2945	0.7696
			**Test Scale**	0.2561	0.7560

Survey question #24 (Q74) among all correlations exhibited the lowest value (−0.009), suggesting potential distortion of the overall measurement. Subsequent analysis after excluding Q74 resulted in a slight increase in the alpha coefficient to 0.79 (95% CI: 0.59, 0.98) and an increase in average inter-item correlation of 0.32 (95% CI: 0.09, 0.54). The analysis of omitted items displayed consistent coefficients, ranging from 0.69 to 0.79.

### Creation of a worry score and types of worriers

**Fig 1:**
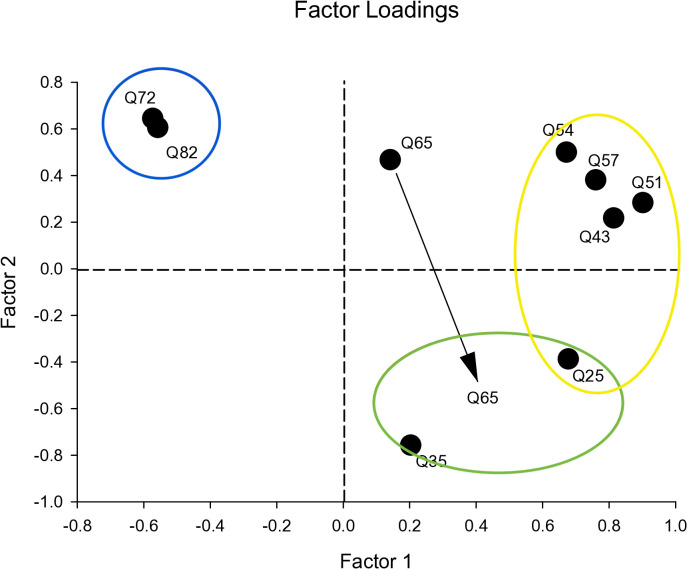
A model with factor loadings displaying three factors after latent factor analysis with varimax rotation. Physicians fell into three clusters of distinct worrier types. Factor 1 or Yellow = concerns about patient safety and occupational harmony. Factor 2 or Green = desire to self-preserve and negatively cope. Factor 3 or Blue = concerns about personal safety and their own mortality potential. Question numbers, as outlined in Table 4, are shown for each of the factor classifications. Q25 represents those who experienced burnout during COVID-19. Q65 represents a reliance on positive coping mechanisms, but has a negative sign belonging to Factor 2, as indicated by the arrow.

**Fig 2:**
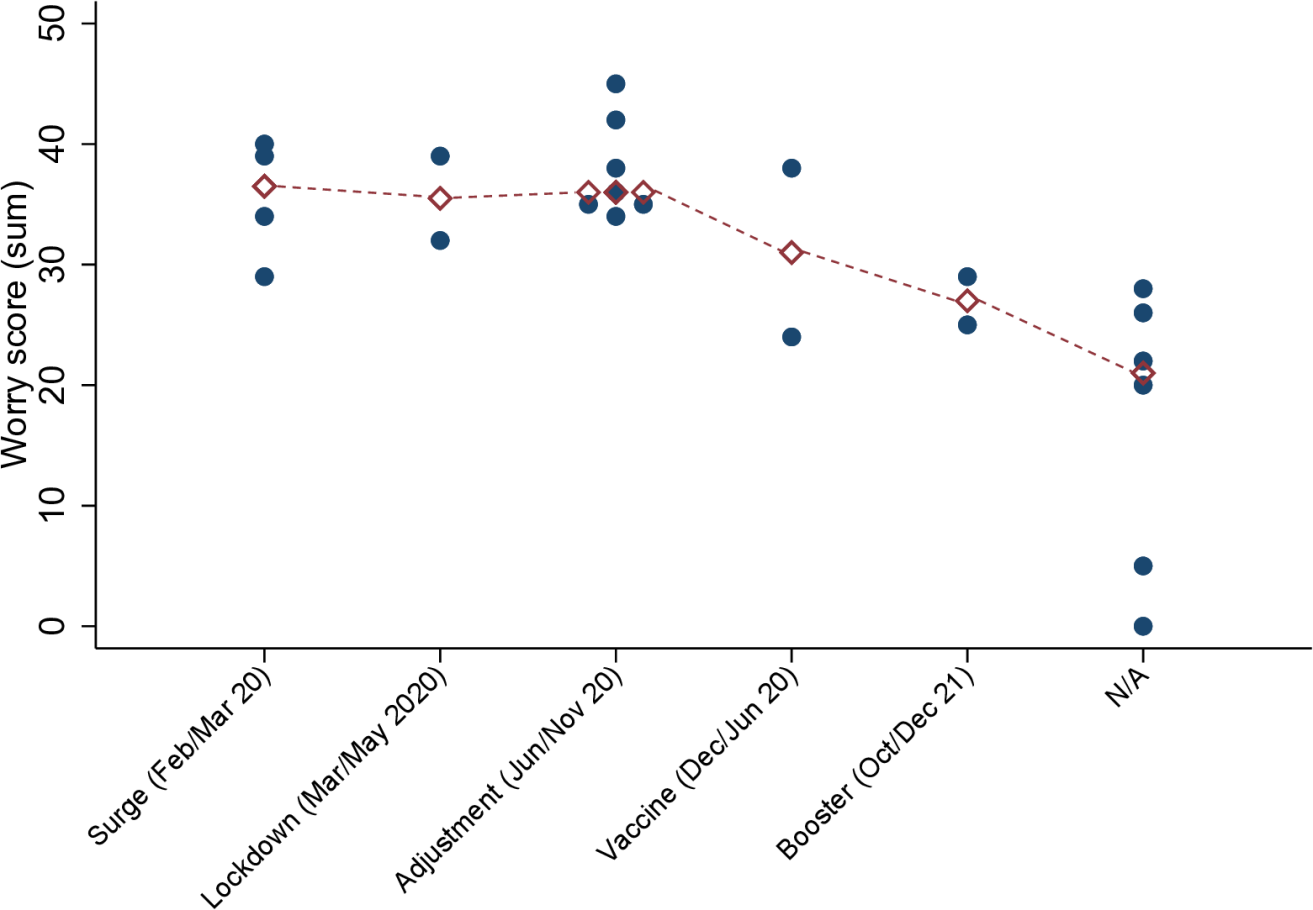
Worry score associated with Time Course Selection during the COVID-19 time period. Physicians felt the most burnout during the Adjustment Period from June 2020 – November 2020. Red diamonds are the determined median values for each group. Worry Scores analyzed by Time Course Period showed clear differences by group (Kruskal-Wallis, p = 0.01). The first four time periods were statistically different from the same time periods among physicians who did not experience burnout (indicated as N/A on the graph).

We sought to develop a Worry Score that incorporated three factors from the survey data: [1] patient safety and occupational harmony, [2] negative coping, and [3] personal safety. We evaluated the uniqueness in the variance shared with survey questions for each factor, where lower uniqueness values were relevant for the items in each of the three factors. Uniqueness represented the proportion of variance not accounted for by the extracted factors. In other words, the more unique a question was, the lower the chance was that it would not fit with or be categorized as one of the three factors in the Worry Score. For example, a uniqueness value of 0.1 indicated that only 10% of the variability in the variable was unique and not explained by the factors. Conversely, a value of 0.7 suggested that 70% of the variability in that variable was unique to that variable and was not explained by the underlying factors identified in the analysis. We found that 9 questions from the survey provided unique relationships for factors (Table 3) supporting the structure of the extracted factors and indicating different types of worry. The latent factor analysis showed that both Q74 and Q82 stood in opposition to Q43. Additionally, Q65 was in opposition to both Q25 and Q35, but still significant enough to potentially include it under Negatively Coping (Factor 2, Green), with note of a weaker correlation (−0.4326) and high uniqueness value (0.7053). Additional data is needed to decide for Q65, as it could present as its own unique factor with additional questions added to the analysis. Based on latent factor analysis with varimax rotation, we identified that physicians displayed 3 distinct worry types or factors (Fig 1). Worry types included: (Factor 1, Yellow) physicians with a desire to self-preserve but showed negative coping, (Factor 2, Green) physicians with a desire for occupational harmony but with concerns about patient safety, and (Factor 3, Blue) physicians with concerns about personal safety and their mortality potential. Thus, we utilized the sum of scores for the nine questions (Table 4) to develop a Worry Score for each study participant (max of 45). We found that the Worry Score fluctuated over the course of the COVID-19 time period (Fig 2). Specifically, Worry Scores for time periods before the rollout of the booster vaccine were significantly higher in physicians with self-reported burnout compared to physicians who did not experience burnout (p = 0.01). In addition, physicians demonstrated the highest Worry Scores during the adjustment period (June 2020 – November 2020), likely due to ongoing uncertainties and adjustments requires as the initial surge of cases stabilized but new challenges emerged.

**Table 4:** Structure of Factors After Varimax Rotation.

Variables and Factor Groupings	Factor 1 Yellow	Factor 2 Green	Factor 3 Blue	Uniqueness Score
**Factor 1 – Yellow – Patient Safety and Occupational Harmony**				
Survey #12 (Q51) On a scale of 1–5, how much did you feel MOST affected in your ability to ensure patient safety during the selected Time Course Period?	0.9281			0.0879
Survey #15 (Q54) On a scale of 1–5, how much did you feel MOST NEGATIVELY affected the quality of care you provided to patients during the selected Time Course Period?	0.8433			0.2826
Survey #18 (Q57) On a scale of 1–5, how much did you feel MOST NEGATIVELY affected in your relationships, personal or work-related during the selected Time Course Period?	0.8718			0.2269
Survey #9 (Q43) On a scale of 1–5, how much did you feel MOST negatively affected in your typical work life during the selected Time Course Period?	0.7479	−0.4787		0.1766
**Factor 2 – Green – Negatively Coping**				
Survey #24 (Q74) On a scale of 1–5, how often did you find yourself relying on negative coping mechanisms during the selected Time Course Period?		0.9568		0.0384
Survey #27 (Q82) On a scale of 1–5, how strong was your desire to leave the field of medicine during the selected Time Course Period?		0.9443		0.0785
Survey #21 (Q65) On a scale of 1–5, how often did you find yourself relying on positive coping mechanisms during the selected Time Course Period?		−0.4326		0.7053
**Factor 3 – Blue – Personal Safety**				
Survey #3 (Q25) On a scale of 1–5, how much did you feel MOST burnt out during the selected Time Course Period?	0.4562		0.8080	0.0998
Survey #6 (Q35) On a scale of 1–5, how much did you feel MOST concerned about your safety during the selected Time Course Period?			0.9092	0.1138

Note: only correlations greater than 0.4 are shown.

### Coping mechanisms and strategies to address perceived burnout

**Fig 3:**
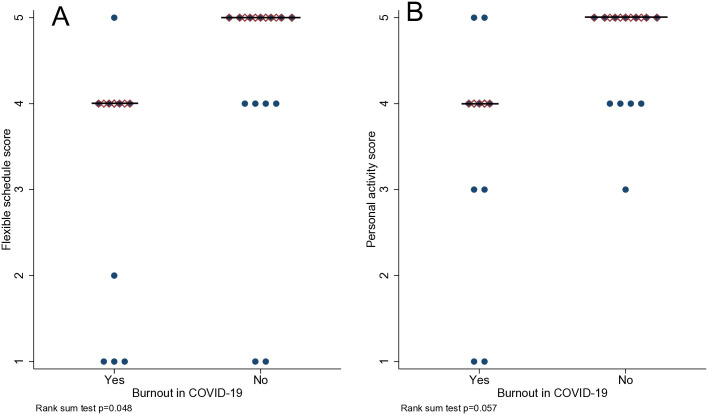
Ranking of flexible schedule and personal wellness activities relieving burnout in RGV physicians. **(A)** During COVID-19, both physician groups who did and did not experience burnout ranked Flexible Schedule significantly higher than other means of interventions to mitigate and relieve burnout experience. Flexible Schedule scores were perceived as beneficial in coping with burnout (p = 0.048). **(B)** In addition, both physician groups who did and did not experience burnout ranked Personal Wellness Activities significantly higher than other means of interventions to mitigate and relieve burnout experience. Personal Wellness Activity scores showed a benefit in coping with burnout (p = 0.041).

As the questionnaire (Appendix A) outlined, all physicians self-reported burnout. We found that during COVID-19, regardless of feelings of burnout, physicians ranked flexible schedules significantly higher than other means of interventions to relieve burnout experience (p = 0.048) (Fig 3A). In addition, personal wellness activities were found to mitigate and relieve burnout experience regardless of feelings of burnout (p = 0.0411) (Fig 3B).

## Discussion

This pilot study developed a novel survey and score to measure the perceived burnout status in healthy medical personnel during the pandemic in a rural and medically underserved area. The Worry Score included the stressors faced by physicians practicing in rural settings during the COVID-19 pandemic, offering a nuanced understanding of concerns about personal safety, strategies to cope with burnout, and occupational alternatives. The COVID-19 pandemic exposed many challenges of the American medical system, including physician burnout. This issue not only harms individual physicians, but also impacts patients, coworkers, and families through a trickle-down mechanism, where the well-being of the healthcare providers directly impacts the quality of care and overall healthcare system efficacy [3,4,30,33,38,50]. The true cost of physician burnout has not fully been measured, but certainly impacts many areas, with reports of worsened patient outcomes, longer recovery times, and increased healthcare costs due to higher rates of medical errors and staff turnover [15,16,18,24,29,35,36,41,50,53,56,75,85]. Education and awareness of physician burnout have been on the rise recently, with initiatives such as resilience training programs, mental health support services, and institutional changes aimed at reducing workload [86,87]. The COVID-19 pandemic has acted as a call to action for all those who participate in our medical system, highlighting the urgent need for systemic solutions.

Our study aimed to measure the rate of burnout in physicians practicing in the RGV, an area already home to significant medical disparities and lack of healthcare access [54,57,88–93]. We found that of the 31 physicians surveyed, 63% experienced burnout at some point during their career, with 43% experiencing burnout during the COVID-19 pandemic. Through our collected data, we created a novel tool to assess responses to burnout, particularly in rural settings. The Worry Score tool classifies responses regarding burnout into three categories: those that used negative mechanisms to cope, those primarily concerned with personal safety, and those most concerned with patient safety; with the latter two categories involved in using positive coping mechanisms. Additionally, we asked physicians to rank specific burnout interventions on a Likert scale and found that flexible scheduling and time for personal wellness activities were the most beneficial to our study group. Our findings are in alignment with other studies that have suggested organizational efforts such as reducing workload, optimizing clinical staff, and implementing peer support programs are effective at reducing burnout [13,37,44,45,94,95].

In recent years, the medical community has become more diverse and copes with stressors in varying ways. Our Worry Score identified three distinct groupings when analyzing responses regarding coping mechanisms during the COVID-19 pandemic. Group one relied on negative coping mechanisms and was most concerned with self-preservation. Groups two and three both relied on positive coping mechanisms, with the prior primarily concerned with personal safety and the latter concerned with patient safety. These groupings suggest that physicians are human beings with individual motivations and desires, which must be addressed when discussing systemic burnout interventions. A French study conducted in 2020 used a similar analysis model to find protective and harmful personality traits for burnout in future neurosurgeons [85]. Overall, the Worry Score we created has comparable potential in identifying physicians at risk of burnout, particularly in rural areas, and may help administrative and program departments better support physician well-being.

Combating burnout using wellness practices is essential to the health and well-being of physicians and the patients they treat. Our study aimed to assess how important specific wellness interventions (flexible schedules, wellness check-ins/surveys/education, workplace wellness activities, personal wellness activities, and mental health services) were to physicians practicing in the RGV during the COVID-19 pandemic. When asked to rank on a Likert scale of 1–5 how beneficial each wellness activity was to their mental and physical health, RGV physicians chose to have a flexible schedule and personal wellness activities as the most impactful interventions (Fig 3). Other studies have also found that physicians who engage in wellness activities in their free time have less burnout compared to their peers [9,42,45,56,96]. Our data suggests that for healthcare systems in similar environments as the RGV, it may be essential to combat burnout in their workplaces and improve patient outcomes using flexible schedules and personal wellness activities. However, while personal wellness interventions may be beneficial on an individual level, they are a response to a much larger problem that will require a systems-based model to eliminate [13,19,41,56,94]. Researchers at the Pennsylvania State School of Medicine have developed The Health Professionals Hierarchy of Needs that aims to simplify healthcare worker needs so leaders may better care for their staff, and thus, their patients [35]. The identification and alleviation of burnout is essential, but still follows the typical American model of healthcare- treating the symptoms instead of preventing the disease. With the use of our Worry Score tool, healthcare systems may be able to identify physicians at risk of burnout and intervene early, addressing the issue before it even occurs. The Worry Score tool may also be a helpful adjunct to current systems levels practices, such as resiliency training, mindfulness and stress management systems. As the American healthcare system continues to develop and shift to huge hospital systems and conglomerate-owned practices, information on individual and organization-wide burnout reduction strategies becomes ever more vital.

Our study assessed burnout specifically during the COVID-19 pandemic (December 2019-June 2021) in the RGV as it placed a significant amount of additional stress on an underserved population. The RGV is a border community of approximately 1.4 million people with an undocumented immigrant population estimated to range in the 100,000’s as of 2019 [89,97]. Approximately 50% of the population does not have health insurance and almost 30% cannot afford to see a doctor [57,89,92,97]. These are significant barriers to care and place many patients in vulnerable situations regarding their health. The COVID-19 pandemic acutely worsened access to healthcare, not just in the RGV but worldwide [51]. Patients were no longer able to see physicians in person, and for many in the RGV telemedicine was not an option due to lack of technology and internet access [9,55,90,91]. Additionally, 45% of the RGV population is obese and 18% is diabetic, both conditions which place patients in an immunosuppressed state [89]. Concurrently, we found that 43% of the 31 RGV physicians surveyed experienced burnout during the COVID-19 pandemic. Given the negative effect burnout has on patient outcomes in addition to the many healthcare barriers already in place in the RGV, it is of the utmost importance to our community to identify potential burnout and design our system to alleviate that burden. However, RGV physicians are not alone, as an international meta-analysis of 45 observational studies found that the prevalence of physician burnout between December 2019 and May 2022 was 54.60% [73]. The COVID-19 pandemic was a source of significant burnout for physicians all over the world. Our healthcare systems will continue to feel this effect until we take steps to alleviate and prevent burnout.

While our study is novel and highlights several components relevant to RGV physician burnout, we acknowledge several limitations. First, all this is a cross-sectional study design and self-reported rankings, limiting the causal effects. Second, because it was a pilot study, we did not use other burnout tools to contribute to a convergent validation of the questionnaire. However, we addressed the known-groups validation instead, obtaining sensitive results. Another limitation was M.D. physicians reached were those involved in university clinics and hospitals to varying degrees, and thus bias may have been introduced by not including a wider range of community physicians, locum tenens physicians, or those who may have retired or were no longer affiliated with the university at the time of data collection. Another additional contributor to bias may have been the lack of inclusion and capture of burnout in other varieties of healthcare workers (e.g., Doctor of Osteopathic Medicine [D.O.], Certified Physician Assistant [PA-C], Advanced Practice Registered Nurse-Nurse Practitioner [APRN-NP], Bachelor of Science in Nursing Registered Nurse [BSN RN]). In addition, here we chose to create a unique questionnaire that would target areas of burnout and coping in the RGV area. Our survey included elements from other standardized burnout surveys [58–65], but we acknowledge the use of a novel survey tool. Development of a novel survey and tool did allow us to identify unique challenges to our population but required substantial review prior to implementation. Our statistical analysis suggests that our questionnaire demonstrated good internal consistency and reliability, comparable to other published survey instruments. However, a limitation of this study is that it did not test convergent and discriminant validity against an established questionnaire.

The purpose of this study was to develop a survey and tool that could assess the prevalence of physician burnout in rural populations. Our study evaluated RGV physicians directly and identified protective and negative factors that contributed to burnout. Our findings showed that a significant proportion of physicians in the RGV experienced burnout, with most encountering it during the adjustment period of June to September 2020. A Worry Score tool was created to measure individuals’ responses to stress and was used to find distinct types of coping mechanisms. The Worry Score is a novel tool to assess how individual physicians respond to burnout and can be used as a preventative measure within healthcare systems. Our study also evaluated ways to alleviate burnout and found the most merit in flexible scheduling and personal wellness activities. Few studies have assessed the prevalence of burnout in underserved areas, and our study is the first to evaluate RGV physicians. Larger surveys should be taken of RGV physicians, with more focus on community physicians, as the majority of our responses came from university-based physicians. Also, future studies include an established burnout instrument to reinforce the validation. Also, future studies should be expanded beyond the RGV and include other healthcare workers to obtain more generalizable data. We suggest that the Worry Score be used in other situations, such as during natural disasters or in times with no additional stressors, to assess its applicability beyond the COVID-19 pandemic.

## Supporting information

S1 Table Descriptive statistics for each Worry Score item. The survey items’ responses varied because participants were not required to answer all items. Q65 was reverse-coded for scoring because conceptually it is a positive coping mechanism. ** Represents the sum of all available items from those with perception of burnout. ***Total Score of all 31 participants.
(DOCX)

S1 Data RawDataBurnoutProject. (XLSX)

S1 File SurveryBurnoutProject. (PDF)
