# Supplementary material for: Development of a survey and worry score to evaluate physician burnout and wellness interventions during COVID-19 in the Rio Grande Valley: A pilot study
Source: PLoS One. 2026 Mar 20;21(3):e0342993. doi: 10.1371/journal.pone.0342993 (PMC13004364; doi:10.1371/journal.pone.0342993)
Supplement: S1 Table — The survey items’ responses varied because participants were not required to answer all items. Q65 was reverse-coded for scoring because conceptually it is a positive coping mechanism. ** Represents the sum of all available items from those with perception of burnout. ***Total Score of all 31 participants. (DOCX) [file pone.0342993.s001.docx]

Supplementary Table 1.

| Variable | n (% response) | Mean (SD) | Median (min, max) |
| --- | --- | --- | --- |
| Survey #3 (Q25) On a scale of 1-5, how much did you feel MOST burnt out during the selected Time Course Period? | 18 (58%) | 4.2 (1) | 4 (1, 5) |
| Survey #6 (Q35) On a scale of 1-5, how much did you feel MOST concerned about your safety during the selected Time Course Period? | 21 (68%) | 4.4 (1) | 5 (1, 5) |
| Survey #9 (Q43) On a scale of 1-5, how much did you feel MOST negatively affected in your typical work life during the selected Time Course Period? | 21 (68%) | 4.2 (0.7) | 4 (3, 5) |
| Survey #12 (Q51) On a scale of 1-5, how much did you feel MOST affected in your ability to ensure patient safety during the selected Time Course Period? | 18 (58%) | 4.5 (0.5) | 4.5 (4, 5) |
| Survey #15 (Q54) On a scale of 1-5, how much did you feel MOST NEGATIVELY affected the quality of care you provided to patients during the selected Time Course Period? | 18 (58%) | 4.3 (0.7) | 4 (3, 5) |
| Survey #18 (Q57) On a scale of 1-5, how much did you feel MOST NEGATIVELY affected in your relationships, personal or work-related during the selected Time Course Period? | 19 (61%) | 4.4 (0.6) | 4 (3, 5) |
| Survey #21 (Q65) On a scale of 1-5, how often did you find yourself relying on positive coping mechanisms during the selected Time Course Period?* | 21 (68%) | 4.2 (0.8) | 4 (3, 5) |
| Survey #24 (Q74) On a scale of 1-5, how often did you find yourself relying on these negative coping mechanisms during the selected Time Course Period? | 17 (55%) | 3.6 (1.4) | 4 (1, 5) |
| Survey #27 (Q82) On a scale of 1-5, how strong was your desire to leave the field of medicine during the selected Time Course Period? | 13 (42%) | 3.7 (1.7) | 4 (1, 5) |
| Score only in those with perception of burnout ** | 21 (68%) | 31.5 (9.2) | 34 (5, 45) |
| Total Worry Score *** | 31 (100%) | 22.4 (16.4) | 28 (0, 45) |
