# Supplementary material for: Development of a survey and worry score to evaluate physician burnout and wellness interventions during COVID-19 in the Rio Grande Valley: A pilot study
Source: PLoS One. 2026 Mar 20;21(3):e0342993. doi: 10.1371/journal.pone.0342993 (PMC13004364; doi:10.1371/journal.pone.0342993)
Supplement: S1 File — (PDF) [file pone.0342993.s003.pdf]

Demographics

5) Age (years)

____

6) Ethnicity

- ◯ Caucasian
- ◯ African American
- ◯ Hispanic or Latino
- ◯ Not Listed
- ◯ Prefer not to answer

7) Sex

- ◯ Female
- ◯ Male
- ◯ Nonbinary
- ◯ Prefer not to answer

8) Marital Status

- ◯ Unmarried
- ◯ Married
- ◯ Divorced
- ◯ Widowed
- ◯ Prefer not to answer

9) Number of years in practice, including residency/fellowship training

____

10) Specialty

- ◯ Family
- ◯ Pediatrics
- ◯ Internal Medicine
- ◯ OB/GYN
- ◯ Emergency Medicine
- ◯ Radiology
- ◯ Dermatology
- ◯ Surgery (includes surgical subspecialties)
- ◯ Other
- ◯ Prefer not to answer

11) If you selected "Other" above, please list your specialty here:

____

12) Workplace Setting

- ◯ Hospital-employed
- ◯ Private practice
- ◯ Academia
- ◯ Prefer not to answer

13) What type of Undergraduate Medical Education did you complete?

- ◯ US MD medical school
- ◯ US DO medical school
- ◯ International Medical Graduate (IMG)
- ◯ Prefer not to answer

Burnout

Burnout Definition:

Burnout is a long-term stress reaction marked by emotional exhaustion, depersonalization, and a lack of sense of personal accomplishment. Burned-out physicians are more likely to leave practice, which reduces patients' access to and continuity of care. Burnout can also threaten patient safety and quality of care when depersonalization leads to poor interactions with patients. Additionally, physicians experiencing burnout can suffer from impaired attention, memory, and executive function

Using the definition of burnout found above, please answer the following questions.

1) According to the previous definition, have you ever experienced burnout in your medical career?
   - ◯ Yes
   - ◯ No

2) In the two-year timespan ranging from January 2020 to December 2021, did you experience burnout in your medical career?
   - ◯ Yes
   - ◯ No

Survey

Burnout Survey Questions

1) 1) At what point during the COVID-19 pandemic were you feeling MOST burnt out?

- ◯ 1st surge (Feb - Mar 2020)
- ◯ Lockdown (Mar-May 2020)
- ◯ Adjustment Period (June 2020-November 2020)
- ◯ Vaccine Distribution (December 2020-June 2021)
- ◯ Delta variant (June 2021-September 2021)
- ◯ Booster available (October 2021-December 2021)
- ◯ N/A

2) In your opinion and per your selected time period, what do you attribute this feeling to? Select all that apply.

- ☐ PPE supply shortage
- ☐ No Vaccine protection
- ☐ No treatment
- ☐ Lack of hospital beds
- ☐ Personal Safety
- ☐ Lack of Support by local/state politicians
- ☐ Other
- ☐ N/A

	1	2	3	4	5	N/A
3) On a scale of 1-5, how much did your selected answer choice(s) influence this feeling? (1= least, 5 = most)	◯	◯	◯	◯	◯	◯

4) 2) At what point during the COVID-19 pandemic were you feeling MOST concerned about your safety?

- ◯ 1st surge (Feb - Mar 2020)
- ◯ Lockdown (Mar-May 2020)
- ◯ Adjustment Period (June 2020-November 2020)
- ◯ Vaccine Distribution (December 2020-June 2021)
- ◯ Delta variant (June 2021-September 2021)
- ◯ Booster available (October 2021-December 2021)
- ◯ N/A

5) In your opinion and per your selected time period, what do you attribute this feeling to? Select all that apply.

- ☐ PPE supply shortage
- ☐ No Vaccine protection
- ☐ No treatment
- ☐ Lack of hospital beds
- ☐ Personal Safety
- ☐ Lack of Support by local/state politicians
- ☐ Other
- ☐ N/A

	1	2	3	4	5	N/A
6) On a scale of 1-5, how much did your selected answer choice(s) influence this feeling? (1= least, 5 = most)	◯	◯	◯	◯	◯	◯

7) 3) At what point did the COVID-19 Pandemic MOST negatively affect your typical work life? (Examples include but are not limited to: faster pace, chaotic environment, etc.)

- ◯ 1st surge (Feb - Mar 2020)
- ◯ Lockdown (Mar-May 2020)
- ◯ Adjustment Period (June 2020-November 2020)
- ◯ Vaccine Distribution (December 2020-June 2021)
- ◯ Delta variant (June 2021-September 2021)
- ◯ Booster available (October 2021-December 2021)
- ◯ N/A

8) In your opinion and per your selected time period, what do you attribute this negative effect on your typical work life to? Select all that apply.

- ☐ PPE supply shortage
- ☐ Staff shortages
- ☐ Decreased patient load
- ☐ Increased patient load
- ☐ Other
- ☐ N/A

9) On a scale of 1-5, how much did your selected answer choice(s) negatively affect your typical work life? (1= least, 5 = most)

1	2	3	4	5	N/A
◯	◯	◯	◯	◯	◯

10) 4) At what point did the COVID 19 Pandemic MOST affect your ability to ensure patient safety? (Examples of poor patient safety include, but are not limited to: medication errors, healthcare-associated infections, unsafe surgical care procedures, diagnostic errors, etc.)

- ◯ 1st surge (Feb - Mar 2020)
- ◯ Lockdown (Mar-May 2020)
- ◯ Adjustment Period (June 2020-November 2020)
- ◯ Vaccine Distribution (December 2020-June 2021)
- ◯ Delta variant (June 2021-September 2021)
- ◯ Booster available (October 2021-December 2021)
- ◯ N/A

11) In your opinion and per your selected time period, what do you attribute this effect on patient safety to? Select all that apply.

- ☐ PPE supply shortage
- ☐ No Vaccine protection
- ☐ No treatment
- ☐ Staff shortages
- ☐ Other
- ☐ N/A

12) On a scale of 1-5, how much did your selected answer choice(s) affect patient safety? (1= least, 5 = most)

1	2	3	4	5	N/A
◯	◯	◯	◯	◯	◯

13) 5) At what point did the COVID-19 Pandemic MOST NEGATIVELY affect the quality of care you provided to patients? (Quality health care should be patient-centered, equitable, efficient, and timely.)

- ◯ 1st surge (Feb - Mar 2020)
- ◯ Lockdown (Mar-May 2020)
- ◯ Adjustment Period (June 2020-November 2020)
- ◯ Vaccine Distribution (December 2020-June 2021)
- ◯ Delta variant (June 2021-September 2021)
- ◯ Booster available (October 2021-December 2021)
- ◯ N/A

14) In your opinion and per your selected time period, what do you attribute this negative effect on the quality of care to? Select all that apply.

- ◯ PPE supply shortage
- ◯ Staff shortages
- ◯ Increased patient load
- ◯ Other
- ◯ N/A

15) On a scale of 1-5, how much did your selected answer choice(s) negatively affect quality of care? (1= least, 5 = most)

1	2	3	4	5	N/A
◯	◯	◯	◯	◯	◯

16) 6) At what point did you feel the COVID-19 Pandemic MOST NEGATIVELY affected your relationships, personal or work-related?

- ◯ 1st surge (Feb - Mar 2020)
- ◯ Lockdown (Mar-May 2020)
- ◯ Adjustment Period (June 2020-November 2020)
- ◯ Vaccine Distribution (December 2020-June 2021)
- ◯ Delta variant (June 2021-September 2021)
- ◯ Booster available (October 2021-December 2021)
- ◯ N/A

17) In your opinion and per your selected time period, what do you attribute this negative effect on your relationships to? Select all that apply.

- ◯ Increased patient load
- ◯ Finances
- ◯ Personal responsibilities (childcare, quality time, etc)
- ◯ Other
- ◯ N/A

18) On a scale of 1-5, how much did your selected answer choice(s) negatively affect your relationships? (1= least, 5 = most)

1	2	3	4	5	N/A
◯	◯	◯	◯	◯	◯

19) 7) At what point during the COVID-19 Pandemic did you start to rely on POSITIVE coping mechanisms?

- ◯ 1st surge (Feb - Mar 2020)
- ◯ Lockdown (Mar-May 2020)
- ◯ Adjustment Period (June 2020-November 2020)
- ◯ Vaccine Distribution (December 2020-June 2021)
- ◯ Delta variant (June 2021-September 2021)
- ◯ Booster available (October 2021-December 2021)
- ◯ N/A

20) In your opinion and per your selected time period, what type of positive coping mechanisms did you rely on? Select all that apply.

- ☐ Physical Health (yoga, nature walks, etc)
- ☐ Mental & Emotional Health (telehealth medicine, counseling, psychiatric support, etc)
- ☐ New hobbies (cooking, etc)
- ☐ Mindfulness (Meditation, sensory grounding, etc)
- ☐ Other
- ☐ N/A

21) On a scale of 1-5, how often did you find yourself relying on these positive coping mechanisms? (1= least, 5 = most)

1	2	3	4	5	N/A
◯	◯	◯	◯	◯	◯

22) 8) At what point during the COVID-19 Pandemic did you start to rely on NEGATIVE coping mechanisms?

- ◯ 1st surge (Feb - Mar 2020)
- ◯ Lockdown (Mar-May 2020)
- ◯ Adjustment Period (June 2020-November 2020)
- ◯ Vaccine Distribution (December 2020-June 2021)
- ◯ Delta variant (June 2021-September 2021)
- ◯ Booster available (October 2021-December 2021)
- ◯ N/A

23) In your opinion and per your selected time period, what type of negative coping mechanisms did you rely on? Select all that apply.

- ☐ Physical Health (over or under eating, lack of exercise, etc)
- ☐ Mental & Emotional Health (self-harm, lack of therapy, etc)
- ☐ Depressive behavior (withdrawing, loss of interest, etc)
- ☐ Risky or aggressive behaviors (gambling, sex, etc)
- ☐ Recreational drug/alcohol use
- ☐ Other
- ☐ N/A

	1	2	3	4	5	N/A
24) On a scale of 1-5, how often did you find yourself relying on these negative coping mechanisms? (1= least, 5 = most)	◯	◯	◯	◯	◯	◯

25) 9) At what point during the COVID-19 Pandemic did you consider leaving the field of medicine?

- ◯ 1st surge (Feb - Mar 2020)
- ◯ Lockdown (Mar-May 2020)
- ◯ Adjustment Period (June 2020-November 2020)
- ◯ Vaccine Distribution (December 2020-June 2021)
- ◯ Delta variant (June 2021-September 2021)
- ◯ Booster available (October 2021-December 2021)
- ◯ N/A

26) In your opinion and per your selected time period, what factors contributed to your desire to leave medicine? Select all that apply.

- ☐ Lack of control at work (unpredictable hours, patient load, etc)
- ☐ Lack of support at work
- ☐ Burn out
- ☐ Finances
- ☐ Other
- ☐ N/A

	1	2	3	4	5	N/A
27) On a scale of 1-5, how strong was your desire to leave the field of medicine? (1= least, 5 = most)	◯	◯	◯	◯	◯	◯

On a scale of 1-5, rate how each of the following services helped you cope with feelings of burnout, exhaustion, and fatigue during the COVID-19 Pandemic. (1= least, 5 = most)

		1	2	3	4	5	N/A
28)	Flexible Schedule	○	○	○	○	○	○
29)	Wellness surveys and subsequent change(s)	○	○	○	○	○	○
30)	Wellness check-ins from supervisors and/or colleagues	○	○	○	○	○	○
31)	Presentations and educational materials on wellness and burnout recognition	○	○	○	○	○	○
32)	Workplace-coordinated wellness activities	○	○	○	○	○	○
33)	Personal, non-workplace wellness activities	○	○	○	○	○	○
34)	Mental health services	○	○	○	○	○	○
